# An evolutionarily conserved intronic region controls the spatiotemporal expression of the transcription factor Sox10

**DOI:** 10.1186/1471-213X-8-105

**Published:** 2008-10-26

**Authors:** James R Dutton, Anthony Antonellis, Thomas J Carney, Frederico SLM Rodrigues, William J Pavan, Andrew Ward, Robert N Kelsh

**Affiliations:** 1 Centre for Regenerative Medicine, Department of Biology and Biochemistry, University of Bath, Bath, BA2 7AY, UK; 2 Genetic Disease Research Branch, National Human Genome Research Institute, National Institutes of Health, Bethesda, MD, USA; 3 Max-Planck Institute for Immunobiology, Freiberg, Germany; 4 Stem Cell Institute, University of Minnesota, Minneapolis, MN, USA

## Abstract

**Background:**

A major challenge lies in understanding the complexities of gene regulation. Mutation of the transcription factor SOX10 is associated with several human diseases. The disease phenotypes reflect the function of SOX10 in diverse tissues including the neural crest, central nervous system and otic vesicle. As expected, the SOX10 expression pattern is complex and highly dynamic, but little is known of the underlying mechanisms regulating its spatiotemporal pattern. *SOX10 *expression is highly conserved between all vertebrates characterised.

**Results:**

We have combined in vivo testing of DNA fragments in zebrafish and computational comparative genomics to identify the first regulatory regions of the zebrafish *sox10 *gene. Both approaches converged on the 3' end of the conserved 1^st ^intron as being critical for spatial patterning of *sox10 *in the embryo. Importantly, we have defined a minimal region crucial for this function. We show that this region contains numerous binding sites for transcription factors known to be essential in early neural crest induction, including Tcf/Lef, Sox and FoxD3. We show that the identity and relative position of these binding sites are conserved between zebrafish and mammals. A further region, partially required for oligodendrocyte expression, lies in the 5' region of the same intron and contains a putative CSL binding site, consistent with a role for Notch signalling in *sox10 *regulation. Furthermore, we show that β-catenin, Notch signalling and Sox9 can induce ectopic *sox10 *expression in early embryos, consistent with regulatory roles predicted from our transgenic and computational results.

**Conclusion:**

We have thus identified two major sites of *sox10 *regulation in vertebrates and provided evidence supporting a role for at least three factors in driving *sox10 *expression in neural crest, otic epithelium and oligodendrocyte domains.

## Background

Sox10 is an essential transcription factor regulating development of the neural crest, otic vesicle and oligodendrocytes [1-20]. Study of Sox10 mutant phenotypes in mouse and fish, coupled with overexpression and knockdown studies in other vertebrate models, have allowed evaluation of Sox10 function in development (reviewed in [21,22]. It is likely to have distinct roles at different stages, at least in the development of neural crest and its derivatives. These include roles in maintenance of multipotency in neural crest cells, specification of pigment cell and neural fates from the neural crest and differentiation/maintenance of peripheral glial cells. In oligodendrocytes, Sox10 is necessary for differentiation [17]. These roles in development are reflected in the association of SOX10 with several congenital conditions in humans, particularly in Waardenburg-Shah syndrome (OMIM#277580), involving defects in pigmentation and the enteric nervous system [3,23], and in PCWH (OMIM#609136), a complex syndrome involving dysmyelination in the central and peripheral nervous systems with Waardenburg-Shah syndrome [6,24-26]. The recent identification of some Sox10 target genes has, in part, helped clarify these disease phenotypes (reviewed in [21].

To date, these congenital diseases have been associated with changes in the SOX10 coding region, but abnormal regulation of *SOX10 *expression might also be expected to contribute to these diseases. Currently little is known of how expression of *Sox10 *itself is regulated, but the notion that abnormal regulation might underlie human disease conditions is strongly supported by recent work identifying the deletion of a regulatory enhancer of *Sox10 *as causing a weak Waardenburg-Shah-like phenotype in a mouse mutant [27]. It is clear from the detailed descriptions of the expression patterns in human, mouse, chick, frog and zebrafish [4,7,8,10,18,28-33], that regulation of *Sox10 *transcription is likely to be complex, with possibly independent regulation in ear, oligodendrocytes and at different phases of neural crest development. Such a picture has recently also been described for the related gene *Sox9*, where enhancer and promoter elements spread over 300 kb of genomic DNA drive expression in the neural crest, ear and other tissues [34].

Induction and separation of the neural crest from neurectoderm at the neural plate border is described as a multi-step process requiring a tightly regulated temporal cascade of signalling molecules and transcription factors (reviewed in [35,36]. Factors previously implicated in controlling gene expression in the early neural crest include Wnts [37-39], Notch [40,41], FGFs [42], BMPs [43,44], Sox9 [45-47], Snail2/Slug [48], FoxD3 [49-53] and Pax 3 [54]. The first expression of *sox10 *within this cascade appears to be after the initial induction events and hence might be dependent on any of these factors. Furthermore, *sox10 *expression is later seen during phases when neural crest cell maintenance, proliferation and differentiation are ongoing. Expression of *sox10 *in the developing otic epithelium has not been explored, but may be induced by any or all of Sox9, Pax or Dlx, or by signals from the hindbrain e.g. FGF [55-58]. Likewise, *sox10 *expression in the oligodendrocyte lineage may require Notch signalling [59]. Recent studies of *sox10 *regulation in mice has suggested that widely dispersed elements control aspects of *sox10 *expression [27,60,61]. In zebrafish, however, our previous work showed that at least some aspects of early embryonic *sox10 *expression including that in the neural crest can be recapitulated using only relatively proximal promoter sequences [19].

The use of transient and germline transgenic zebrafish are complementary in promoter analysis [62]. Transient transgenics allow very rapid assessment of injected plasmid constructs. However, they are prone to copy number artifacts distorting the level of transgene expression and require cumulative scoring to minimise false negative results and deduce the comprehensive expression pattern of any individual construct tested. Also ectopic expression in areas such as muscle and notochord are common. Alternatively, germline transgenic zebrafish, even when using improved methods that provide higher integration rates [63], still require lengthy raising and identification of adult founders to provide F1 transgenic embryos. However, if multiple lines for each construct are compared to eliminate position effect artefacts the expression profiles in these lines are taken to recapitulate endogenous gene expression.

Given the importance of Sox10 in neural crest development, understanding the regulatory factors driving expression in each tissue will further our understanding of developmental and disease mechanisms and might, for example, suggest further candidate genes underlying Hirschsprung's disease and deafness. Recent advances in sequence analysis programs have allowed identification of regulatory elements in non-coding sequences conserved between both closely related and divergent species [64,65]. Combined with analyses of predicted elements in transgenic embryos such studies enable the functional relevance of predicted regulatory regions to be determined. Nevertheless, some conserved, regulatory regions may show strong sequence divergence and thus may be cryptic, so that a traditional approach of systematic testing of upstream and other potential regulatory regions remains important [66]. We have combined these approaches to begin to determine factors regulating *sox10 *gene expression in embryonic zebrafish. We show that a short 3'region of the zebrafish *sox10 *intron 1 is critical in regulating embryonic sox10 expression and identify an evolutionarily conserved arrangement of binding sites for neural crest-inducing transcription factors clustered within this region.

## Results

### sox10:GFP expression in transient transgenic zebrafish

**Figure 1:**
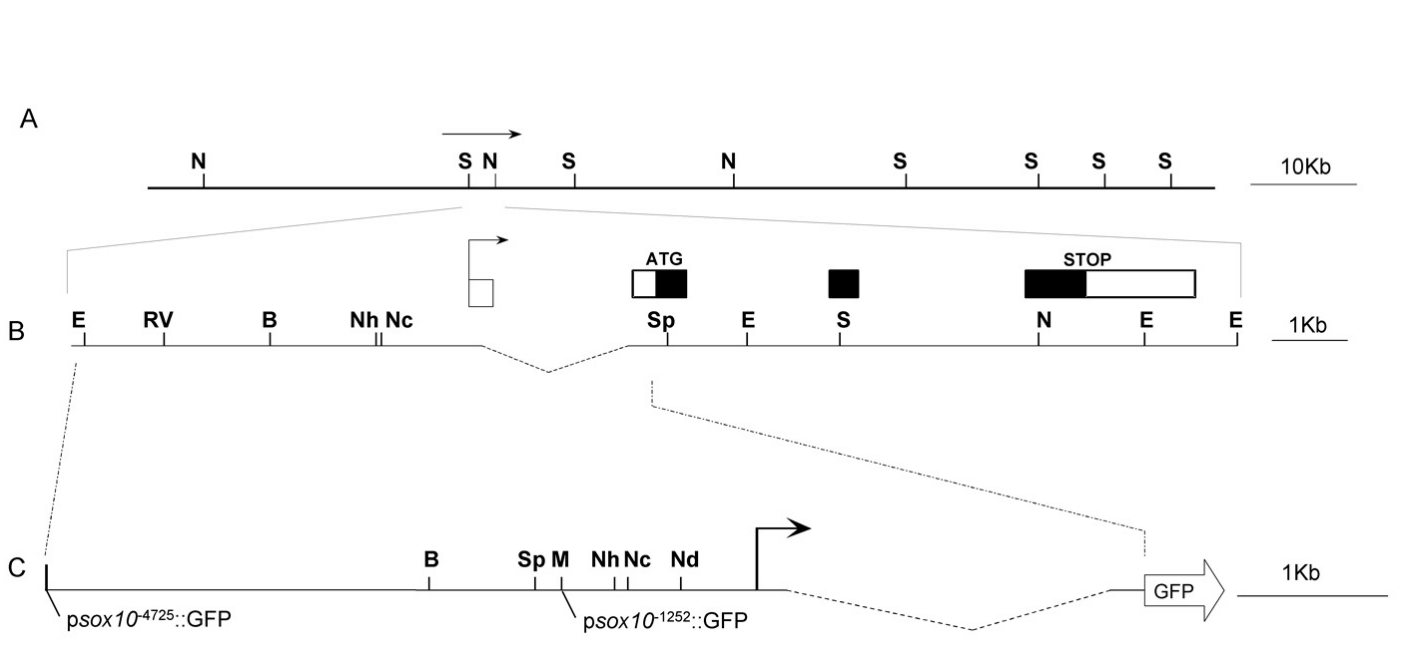
**Genomic context of the zebrafish *sox10 *5' region used in *sox10*:GFP constructs**. A. Restriction map constructed following partial sequencing of PAC BUSMP706I16137Q2 (RZPD) showing the location of DNA encoding *sox10 *(arrow). B. Genomic arrangement of *sox10 *coding region. The bent arrow shows the transcript start position. Closed boxes illustrate coding sequence and open boxes 5' and 3' untranslated regions. C. 5' upstream genomic region of *sox10 *used in construction of *sox10*:GFP plasmids. In B and C a dashed line indicates intron one. Start positions of p*sox10*^-4725^:GFP and p*sox10*^-1252^:GFP are indicated. B *Bam*HI, E *Eco*RI, RV *Eco*RV, N *Not*I, M *Mlu*I, Nh *Nhe*1, Nc *Nco*I, Nd *Nde*I, S *Sma*I, Sp *Spe*I, Sh, *Sph*I.

**Figure 2:**
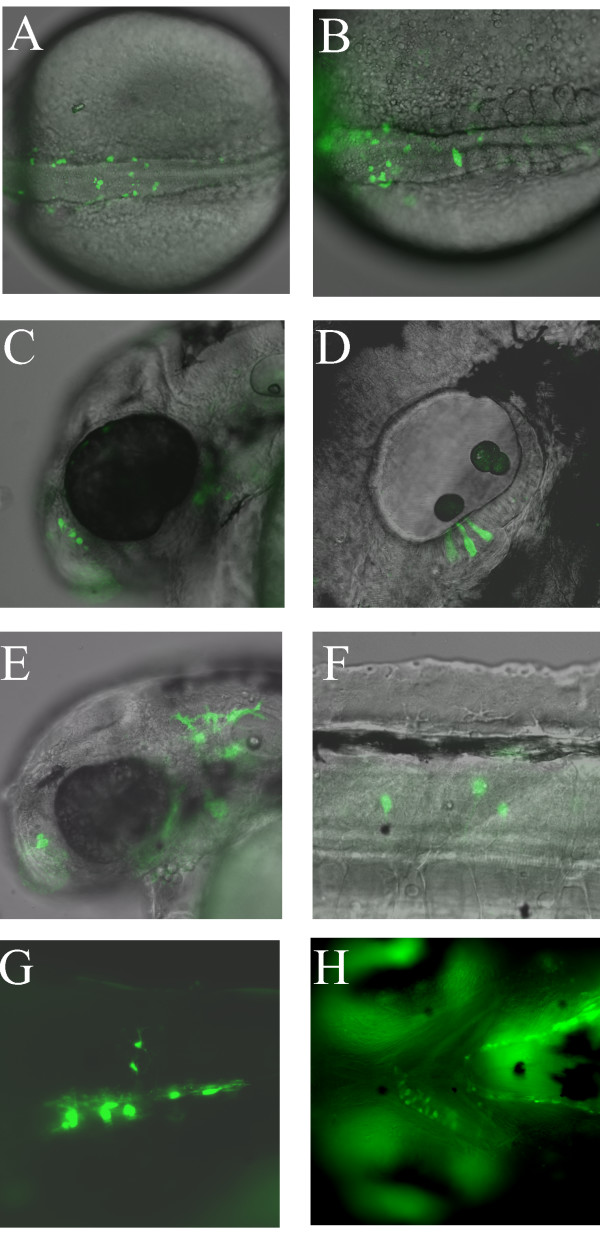
**Examples of mosaic GFP expression from transient transgenic *sox10*:GFP zebrafish injected with p*sox10*^-1252^:GFP plasmid at the 1-cell stage**. A, B. Dorsal views of two separate examples of pre-migratory neural crest GFP expression at the 20-somite stage. C. GFP expression in nasal cells at 48 hpf. D. GFP expression in otic epithelium at 48 hpf. E. GFP expression in xanthophores at 48 hpf. F. GFP expression in interneurons at 48 hpf. G. GFP expression in oligodendrocytes in trunk spinal cord at 96 hpf. H. Jaw cartilage expression at 96 hpf. All panels contain fluorescent images from live embryos (overlaid on DIC images in A-F). Orientation is with anterior to the left, dorsal uppermost except A, B (dorsal) and H (ventral).

To identify functional zebrafish *sox10 *regulatory regions we generated a series of plasmid constructs using 5' genomic DNA flanking the zebrafish Sox10 coding region to drive expression of GFP. Plasmid constructs psox10^-4725^:GFP (containing *sox10 *regulatory regions used previously [13,67]) and psox10^-1252^:GFP contain the *sox10 *gene DNA respectively 4725 bp and 1252 bp immediately 5' of the transcript start site. The latter is defined from our sequencing of zebrafish *sox10 *cDNAs in which the characteristic signature of the 5' 7-methyl-guanosine cap [68] was found immediately 5' to sequences of exon 1 as defined in Fig. 1 (Dutton et al., 2001; TJC, PhD Thesis, University of Bath). Both constructs also contain the zebrafish *sox10 *exon 1, intron 1 and the start of exon 2 (Fig. 1). The *GFP *sequence was placed so the ATG sequence of GFP is in the same relative position as the Sox10 ATG (Fig. 1C). The constructs were then used to test if these regions of DNA contain sufficient regulatory elements to drive detectable GFP expression in a pattern reflecting the endogenous *sox10 *pattern in embryonic zebrafish. When injected into 1-cell zebrafish embryos approximately one third of injected embryos subsequently expressed GFP in one or more discrete cell types. Examples of cells expressing GFP in transient transgenic sox10:GFP embryos are shown in Fig. 2. In embryos injected with constructs psox10^-4725^:GFP or psox10^-1252^:GFP, GFP expression was initially seen at early somite stages in areas corresponding to premigratory neural crest (Fig. 2A, B). By 24 hpf expression was seen in migrating neural crest cells in the head and body. These included migrating cells in all major pathways, including the streams forming the branchial arches, cells populating the peripheral head, and cells on both medial and lateral pathways in the trunk and tail (data not shown). In these transient transgenic embryos some of these cells had the morphology and characteristic pigment of melanophores and xanthophores. GFP expression was also prominent in the olfactory neurons (Fig. 2C), otic epithelium (Fig. 2D) and CNS neurons in both the brain and spinal cord. At 48 hpf GFP expression in xanthophores and melanophores was still visible (Fig. 2E) and Rohon-Beard neurons were sometimes labeled. Cells of the developing inner ear also continued to express GFP strongly. GFP expression at 48 hpf became visible in differentiating cartilage cells of the pectoral fin and the jaw and persisted until at least 7 dpf (Fig. 2H). GFP expression persisted in the interneurons of the CNS for 4 days (Fig. 2F). At 72 hpf GFP expression became visible in developing oligodendrocytes, initially in the ventral neural tube and then in a more widespread pattern as they disperse throughout the CNS (Fig. 2G). GFP expression in lateral line Schwann cells was not seen in transient transgenic fish expressing GFP from the psox10^-1252^:GFP construct but was visible, rarely, in transient transgenic embryos injected with the psox10^-4725^:GFP construct. In addition, GFP expression was seen in notochord and muscle cells, common sites of ectopic reporter expression in transgenic zebrafish [62]. These patterns are fully consistent with those described for constructs containing intermediate length promoter fragments (TJC, PhD Thesis, University of Bath). Together, these data suggested that the genomic *sox10 *DNA region present in all these constructs, but importantly even in the smallest one containing only 1252 bp of upstream sequence, contained regulatory sequences able to drive mosaic GFP expression in transient transgenic zebrafish with an expression profile and timing consistent with that previously described for the zebrafish *sox10 *gene by in situ hybridisation [8].

### Germline sox10:GFP transgenic zebrafish

Before extending the promoter analysis, we checked whether our transient *sox10*:GFP transgenics data would be consistent with the pattern of GFP expression seen in integrated germ line *sox10*:GFP transgenics. We have previously published a similar line made from the p4.9:GFP plasmid, *Tg(-4.9sox10:egfp)*^*ba*2 ^[19], but since this was only a single line it was not clear exactly how representative that pattern might be. Therefore, we generated a number of *sox10*:GFP germ line transgenic zebrafish lines using the psox10^-4725^:GFP and psox10^-1252^:GFP constructs. Multiple transgenic founders were identified for each construct and representative germline transgenic lines were established. Initially 10 founders for sox10^-4725^:GFP and 6 for *sox10*^-1252^:GFP were identified. F2 embryos from all 10 sox10^-4725^*:GFP *founders had similar GFP patterns with no obvious expression domain differences. Lines *Tg(-4725sox10:GFP)*^*ba*3 ^*, Tg(-4725sox10:GFP)*^*ba*4^were subsequently maintained in our fish facility as representative lines although *Tg(-4725sox10:GFP)*^*ba*4 ^embryos have a slightly greater GFP expression level. The *Tg(-1252sox10:GFP)*^*ba*5 ^line was the only one maintained from the shorter construct founders, since it showed no obvious ectopic GFP expression domains; offspring from the other -*1252sox10:GFP *founders sometimes displayed ubiquitous ectopic GFP expression suggesting they were less insulated against insertion position effects.

**Figure 3:**
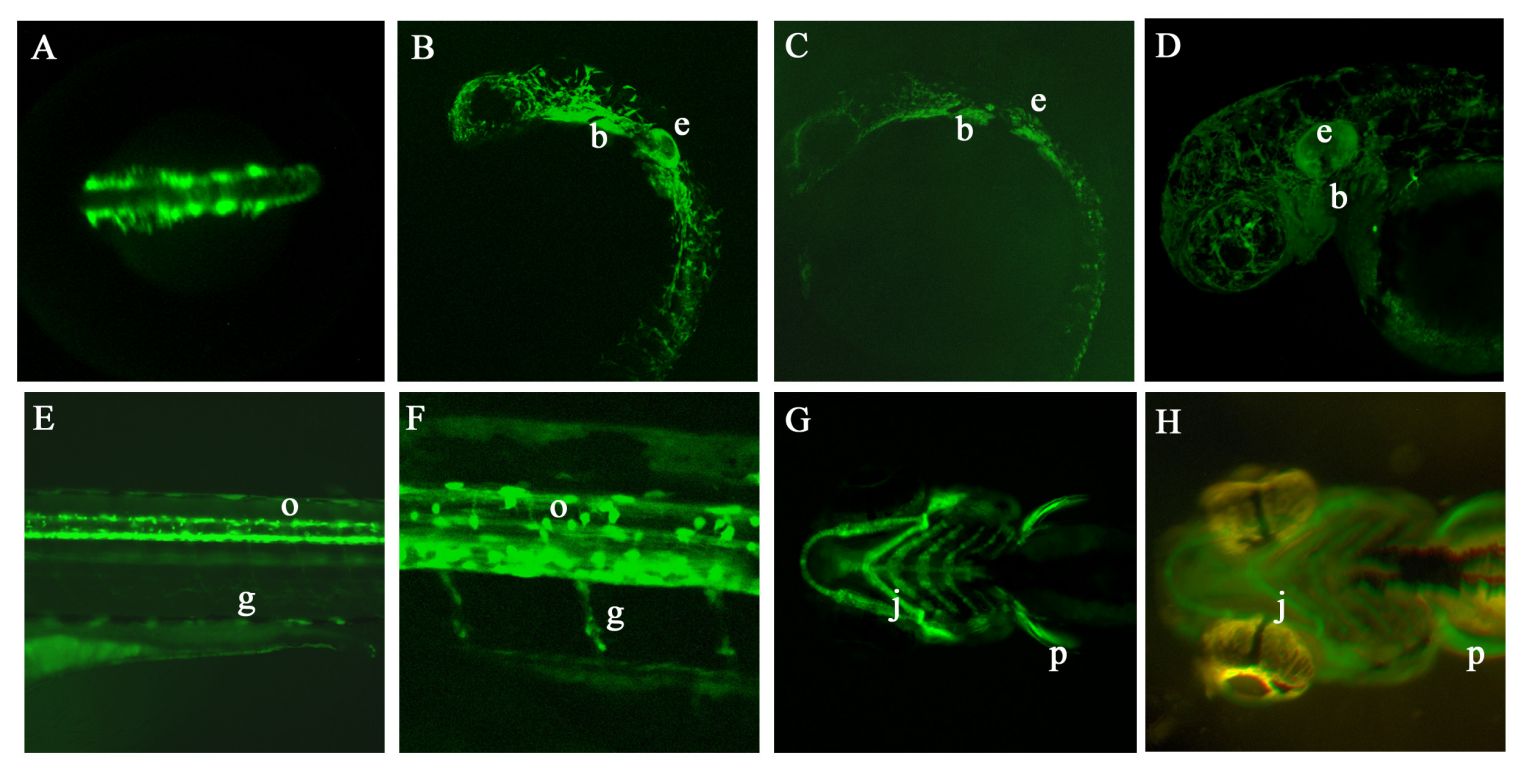
**GFP expression in germline transgenic *sox10:GFP *embryos**. A. Dorsal view of head and anterior trunk of a *Tg(-4725sox10:GFP)*^*ba*4 ^embryo at 16 hpf showing GFP expression in premigratory and early migrating neural crest cells. B, C. Lateral views of transgenic embryos from lines *Tg(-4725sox10:GFP)*^*ba*4 ^(B) and *Tg(-1252sox10:GFP)*^*ba*5 ^(C) to show similarities in GFP expression pattern at 24 hpf. (e ear, b branchial arches). D. Lateral view of head of a *Tg(-4725sox10:GFP)*^*ba*4 ^embryo at 48 hpf showing extensive GFP expression throughout cranial pigment cells, ear (e) and branchial arches (b). E, F. Lateral views of the trunk of *Tg(-4725sox10:GFP)*^*ba*4 ^embryos expressing GFP in oligodendrocytes (arrow) and Schwann cells (arrowhead). G, H. Ventral views comparing GFP expression in the jaw cartilage (j) and pectoral fins (p) of transgenic embryos from *Tg(-4725sox10:GFP)*^*ba*4 ^(F) and *Tg(-1252sox10:GFP)*^*ba*5 ^(G) at 96 hpf.

Transgenic lines *Tg(-4725sox10:GFP)*^*ba*3^, *Tg(-4725sox10:GFP)*^*ba*4 ^and *Tg(-1252sox10:GFP)*^*ba*5 ^showed a pattern of GFP expression consistent with the transient transgenic studies utilising the same DNA constructs. GFP expression in transgenic embryos of lines *Tg(-4725sox10:GFP)*^*ba*4 ^and *Tg(-1252sox10:GFP)*^*ba*5 ^are compared in Table 1 alongside the GFP expression profile of transiently transgenic embryos injected with construct p*sox10*^-1252^:GFP. Examples of GFP expression patterns in *Tg(-4725sox10:GFP)*^*ba*4^and *Tg(-1252sox10:GFP)*^*ba*5 ^are shown in Fig. 3. The earliest GFP expression seen in the *Tg(-4725sox10:GFP)*^*ba*4 ^was in the cranial premigratory neural crest at the 1-somite stage. This expression was not visible in *Tg(-1252sox10:GFP)*^*ba*5^. At 16 hpf GFP expression was visible throughout the premigratory neural crest (Fig. 3A) and by 24 hpf in migrating neural crest cells, branchial arches, ear epithelium and olfactory neurons (Fig. 3B, C). Weak GFP expression was visible in the neural tube. At 48 hpf GFP expression was observed in the ear epithelium, and both melanophores (*Tg(-4725sox10:GFP)*^*ba*4 ^only) and xanthophores were labelled (Fig. 3D), along with jaw and pectoral fin cartilage. GFP expression was seen in both brain and neural tube. Additional sites of reporter expression were seen at 96 hpf when labelled Schwann cells and oligodendrocytes were observed (Fig. 3E, F). GFP expression in pigment cells was largely absent by this time but the jaw and pectoral fin expression remained (Fig. 3G, H). These GFP expression patterns show strong similarities to those we have characterised for intermediate length promoter fragments, although *Tg(-4.9sox10:eGFP)*^*ba*2 ^is unique in showing reduced expression in otic vesicle [19] (TJC, PhD Thesis, University of Bath).

**Table 1:** Spatial expression of GFP in *sox10:GFP *germline transgenic embryos is comparable to that deduced from transiently transgenic embryos

		*Tg(-4725sox10:GFP)*^*ba*4^	*Tg(-1252sox10:GFP)*^*ba*5^	p*sox10*^-1252^:GFP
1-somite	cranial premigratory NC	+/-	-	nd
24 hpf	pre-migratory neural crest	++	+	+
	branchial arches	+++	++	nd
	ear epithelia	+++	++	++
	CNS-brain	-	-	nd
	CNS-neural tube	+	+/-	nd
	olafactory neurons	+++	++	++
	muscle	-	-	nd
	notocord	-	-	nd
48 hpf	ear epithelia	+++	++	++
	Rohon Beard neurons	++	+	++
	Plln Schwann cells	++	+/-	-
	melanophores	+	-	+
	xanthophores	++	+	++
	DRG	++	+	-
	CNS-brain	++	+	+
	CNS-neural tube	++	+	+
	pectoral fin cartilage	+++	++	++
	Jaw cartilage	+++	++	++
	muscle	-	-	+
	notocord	-	-	+
96 hpf	ear epithelia	++	+	+
	DRG neurons	-	-	-
	Oligodendrocytes	+++	+	++
	CNS-brain	+	+	+
	CNS-neural tube	-	-	+
	Jaw cartilage	+++	++	++
	pectoral fin cartilage	+++	++	++
	muscle	-	-	+
	notocord	-	-	+

GFP expression was scored in detail from at least three embryos from each of the *Tg(-4725sox10:GFP)*^*ba*4 ^and *Tg(-1252sox10:GFP)*^*ba*5 ^lines. Embryos were examined at the stages indicated by fluorescent microscopy at 600× and cells expressing GFP scored by position and morphology. The fluorescent signal in each GFP expressing cell type was scored according to the following levels: – none observed, +/- very weak, + weak, ++ good, +++ strong, nd not determined. Data for the p*sox10*^-1252^:GFP construct was deduced from at least 40 transiently transgenic embryos.

Although transgenic offspring from founders injected with the p*sox10*^-1252^:GFP sequence expressed much weaker GFP levels than individual transient transgenics containing the same sequence, the GFP patterns in the transgenic lines were consistent with that deduced from transiently transgenic cohorts injected with p*sox10*^-1252^:GFP. Lines *Tg(-4725sox10:GFP)*^*ba*3 ^and *Tg(-4725sox10:GFP)*^*ba*4 ^expressed GFP at a higher level than *Tg(-1252sox10:GFP)*^*ba*5 ^but the pattern and timing of GFP expression were also comparable in these lines. Some differences in GFP expression between the transgenic lines and the transient transgenic cohorts could be attributed to weak levels of expression in the *Tg(-1252sox10:GFP)*^*ba*5 ^lines. For example, no melanophores expressing GFP were seen in *Tg(-1252sox10:GFP)*^*ba*5 ^embryos although this was seen in the corresponding transient transgenic embryos. Conversely, both glia of the lateral line and DRG associated glia were seen to express GFP in the germline transgenics where this expression had not been seen in the corresponding transient transgenic cohort. This difference presumably reflects the mosaic nature of transgene expression in transients and the consequent low probability of transgene expression in cells derived from uncommon progenitors.

Analysis of the GFP expression pattern in lines *Tg(-4725sox10:GFP)*^*ba*4 ^and Tg*(-1252sox10:GFP)*^*ba*5^, together with our previous transgenic line [19,69], confirmed our conclusion from the transient transgenic analysis that the *sox10 *genomic sequences described contained regulatory sequences able to recapitulate the temporal and spatial pattern of early *sox10 *expression in zebrafish. In addition, however, these data considerably reduce the genomic DNA interval containing these regulatory regions compared with the published *Tg(-4.9sox10:egfp)*^*ba*2 ^line. Consequently, we decided to extend our analysis of zebrafish *sox10 *regulatory regions using a transient transgenic approach.

### Deletion analysis of the zebrafish sox10 promoter

**Figure 4:**
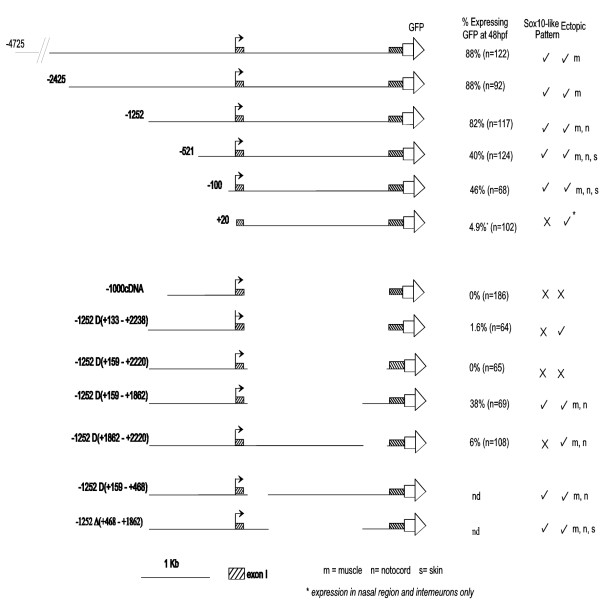
**Deletion analysis of the zebrafish *sox10 *promoter in transient transgenic embryos**. A deletion series of *sox10*:GFP constructs was prepared, with the 5'end of each construct denoted in base pairs 5' of the transcript start (indicated by bent arrows). Please note that -2425*sox10*:GFP here is equivalent to p4.9:GFP [19]. *sox10 *exons 1 and 2 are denoted by shaded boxes, with exon 2 fused to *GFP *(open arrow). Transient transgenic zebrafish were made using each of the *sox10*:GFP constructs shown and all injected fish were examined and any GFP visible by fluorescent microscopy under a dissecting microscope recorded at 48 hpf. Cohorts of GFP positive embryos were further examined under higher power to determine if the GFP expression corresponded to an expected *sox10*-like pattern at 48 hpf and again at 96 hpf. Refer to Table 2 for quantitation of GFP expression by cell-type. m muscle, n notochord, s skin.

In order to identify more precisely where key *sox10 *regulatory elements were located, we created a nested series of 5' zebrafish *sox10 *promoter deletions (Fig. 4). Cohorts of transient transgenic fish were examined for patterns of GFP expression at 48 hpf and 96 hpf points and scored under a fluorescent dissecting microscope at 600× for both numbers of fish expressing any GFP signal and those with GFP signal representing *sox10 *expression (Fig. 4, Table 2). Significantly, the representative *sox10*-like pattern of GFP characterised above was maintained in constructs following increasingly greater 5' deletions. Although the overall level of GFP expression was reduced with larger 5' deletions (as deduced from the lower percentage of fish expressing GFP in any pattern; due to the selection for visible expression the level of GFP expression in individual cells where seen was generally comparable), deletion of the transcript start was required for complete loss of the *sox10*-like pattern of GFP expression. Although expression in fish injected with p*sox10*^-100^:GFP, a construct containing only 100 bp of sequence 5' to the transcript start, resulted in a very low percentage of GFP-expressing fish, the spatial pattern of GFP was generally maintained with all *sox10*-like GFP-positive cell-types represented. This suggested that although control of the level of the *sox10*:GFP expression was mediated by sequences 5' of the transcript start, critical elements regulating the spatial pattern of *sox10 *expression were likely to be located elsewhere within the construct.

**Table 2:** GFP expression in transient *sox10*:GFP zebrafish containing named zebrafish *sox10 *promoter deletions

	**A**					**B**			
	1)	2)	3)	4)	5)	1)	2)	3)	4)

Pigment cells	84.2	80	82.9	15.4	0	90.1	45	66.7	76.2
Ear	81.6	74.2	53.7	28.2	0	70	90	54.2	66.7
Nasal	78.9	91.2	90.2	51.2	0	36.3	15	8.3	33.3
Facial cartilage	72.2	62.5	74.3	30.8	0	74.3	42	45.8	45
Oligodendrocytes	55.6	51.6	16.2	8.8	0	47.1	21	16.7	20
Pectoral fin	44.4	40	39.5	31.4	0	42.4	31.6	25	45
CNS interneurons	89.5	77.1	75.6	56.4	0	72.7	80	75	80.9
Rohon Beard	38.9	25.7	31.7	5.1	0	22.7	35	8.3	28.6
Brain neurons	55.6	65.7	36.5	25.6	0	56.8	70	50	57.1
Heart	16.6	0	9.8	2.9	0	13.6	0	0	9.5
Muscle	19.4	51.4	39	25.6	10	15.9	55	20.8	33.3
Notocord	44.4	37.1	51.2	17.9	18	27.3	0	25	33.3
Embryos scored	n = 38	n = 35	n = 41	n = 39	n = 40	N = 44	n = 20	n = 24	n = 21

A) 1) p*sox10*-1252, 2) p*sox10*-521, 3) p*sox10*-1252Δ(+159–+468, 4) p*sox10*-100, 5) *psox1*-*0*1252Δ(+1862–+2220)

B) 1) p*sox10*-1252Δ(+1862–+2220) + BC^t^, 2) p*sox10*-1252Δ(+1862–+2220) + B, 3) p*sox10*-1252Δ(+1862–+2220) + C, 4) p*sox10*-1252Δ(+1862–+2220) + 2–5

Percentage of embryos positive for GFP in defined region.

Named *sox10*:GFP constructs were injected into 1-cell stage zebrafish embryos. GFP expression in specific cell-types for each embryo was documented at 48 hpf and 96 hpf. Position and morphology of GFP expressing cells were scored at 600× magnification for individual GFP-expressing live embryos selected at random.

To attempt to identify these elements we next constructed plasmids from a zebrafish *sox10 *cDNA (i.e. lacking intron 1), but with the addition of flanking genomic sequences from exon 1 (Fig. 4). The first of these were p*sox10*-1000cDNA:GFP and p*sox10*^-1252Δ(+133–+2220)^:GFP. These both deleted the whole of intron 1 and differed only in 252 bp of DNA 5' of the transcript start. Transgenic fish containing either of these two constructs contained only extremely rare GFP expressing cells and all *sox10*-like expression was absent. This immediately suggested that regulatory elements controlling the *sox10*:GFP expression pattern were present within intron 1. Restriction sites present in intron 1 enabled analysis of constructs containing either 5' or 3' portions of intron 1. Construct p*sox10*^-1252Δ(+1862–+2220)^:GFP deleted 376 bp from the 3' part of intron 1. Transgenics made with this construct showed no GFP expression in a *sox10*-like pattern. In contrast, p*sox10*^-1252Δ(+159–+1862)^:GFP transgenics retained the 3' part of intron 1 and expressed GFP in a *sox10*-like pattern (Fig. 4B). These data suggested that regulatory elements controlling *sox10*:GFP expression were likely to be contained within the 376 bp at the 3' end of intron 1.

### Zebrafish sox10 intron 1 harbors predicted promoter sequences and constrained transcription factor binding sites

**Figure 5:**
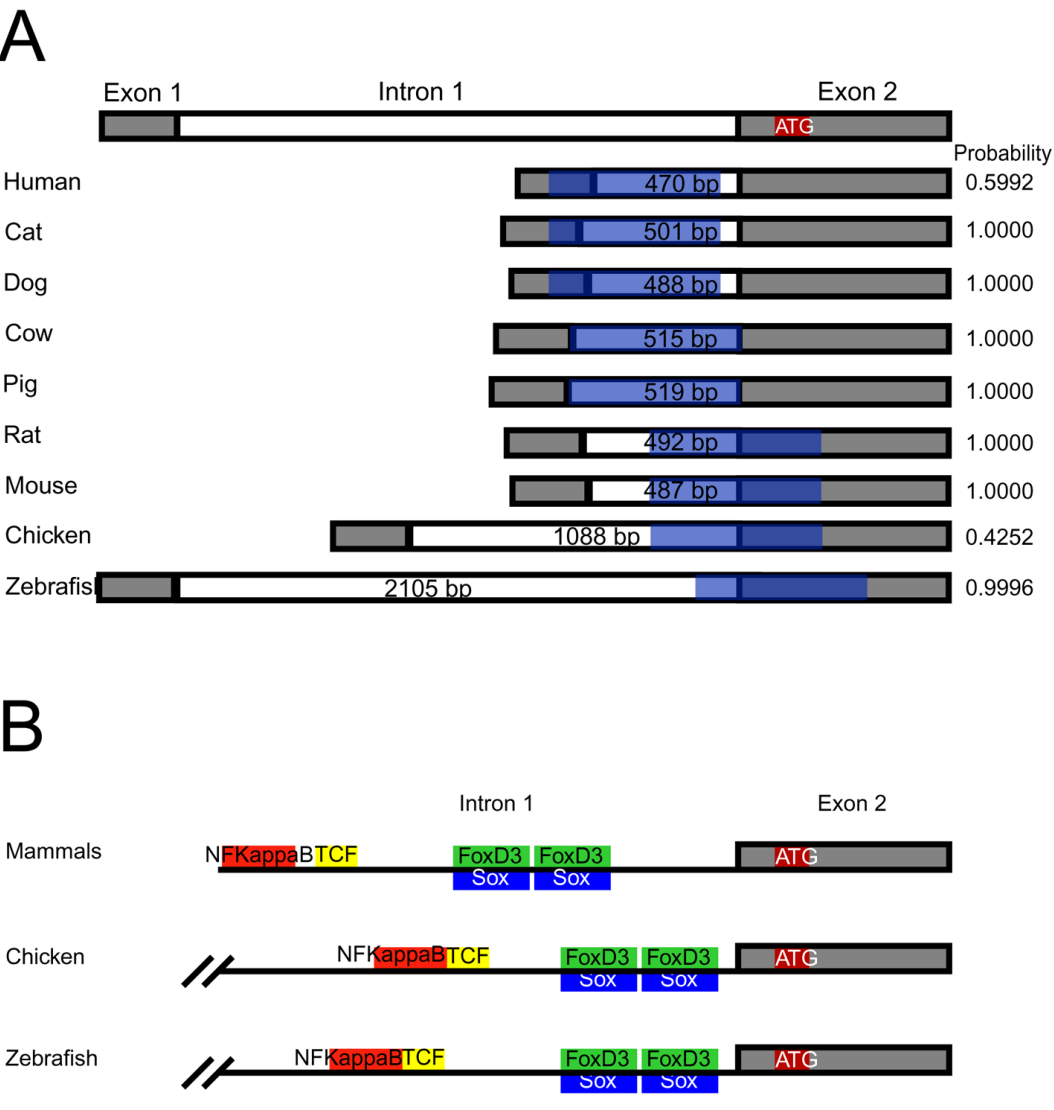
**Promoter and transcription factor binding site predictions at *sox10 *5' genomic sequences**. Genomic sequences from multiple species were used to predict promoter regions (A) and transcription factor binding sites (B) at *sox10 *intron 1. (A) The position and size of intron 1 is indicated for each species. In each case, the promoter predicted by FirstEF is indicated in blue, with the corresponding predicted probability shown on the right. (B) The 3'-most region of *sox10 *intron 1 is shown for mammals, chicken and zebrafish. Highly conserved sequences among mammals were submitted to the TRANSFAC database, as was the entire sequence of intron 1 for chicken and zebrafish. Predictions common to all three are indicated in red (NFKappaB), yellow (Tcf/Lef), green (FoxD3) and blue (Sox family). Note that the prediction and organization relative to the start codon (ATG) of each are conserved between mammals, chicken and zebrafish.

We used the FirstEF promoter prediction algorithm [70] with a view to beginning to identify the location of the *sox10 *promoter in nine species. This software takes a genomic sequence and identifies putative promoters by evaluating genomic regions for CpG islands, promoter regions and first exon donor sites. This algorithm has been shown to appropriately identify about 80% of first exons with a false prediction rate of approximately 15%. In addition to the position of the predictions, FirstEF output includes the probability of finding a true promoter at the predicted location. These analyses revealed that all species have a predicted promoter within intron 1 of the *sox10 *gene (Fig. 5), with results from only two of the eight species (human and chicken) having a probability significantly lower than 1. Interestingly, this software did not predict the transcription start site used in the identified zebrafish *sox10 *cDNAs (AA, data not shown).

Multi-species comparative sequence analysis is emerging as a powerful tool for identifying transcriptional regulatory elements. Indeed, this approach has proven useful for *sox10 *using data from multiple mammalian species [27,60,61]. However, this technique presents unique problems at greater phylogenetic distances and detailed analysis using zebrafish as a reference sequence has not revealed any significant non-coding nucleotide conservation compared to multiple mammalian species or chicken at *sox10 *(data not shown; see also [61]). However, because functionally conserved elements can be missed by these in silico approaches [66], we explored the possibility that smaller motifs (e. g. transcription factor binding sites) are conserved between these species. Specifically, we identified regions 100% identical and at least six basepairs long in seven mammalian species at *sox10 *intron 1 using the ExactPlus software [27]. The conserved fragments were then submitted to TRANSFAC to identify putative transcription factor binding sites. These analyses revealed six biologically-relevant predictions in mammalian species (Fig. 5B; See Additional file 1). To determine if these predictions were conserved in chicken and zebrafish, we submitted the entire intron one sequence for each species to TRANSFAC. Interestingly, chicken and zebrafish sequences have the same predictions as mammals in the 3' most region of intron one. Furthermore, not only are these sequences conserved but so is their arrangement. We hypothesised that these conserved transcription factor binding sites might contribute to an enhancer regulating *sox10 *expression in zebrafish.

### Functional testing of predicted transcription factor binding sites

**Figure 6:**
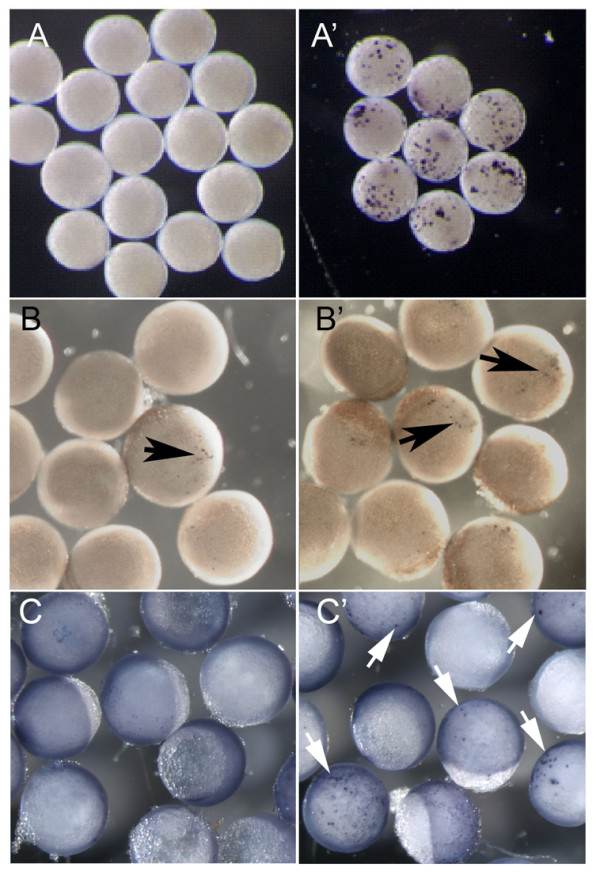
**Functional testing of transcription factors implicated in *sox10 *regulation**. A, B. Early zebrafish embryos (6 hpf), injected with plasmid constructs at the 1-cell stage, were divided and half subjected to a 60 minute 37°C heatshock. All were left to develop for a further 1 hour at 28.5°C before fixing. *sox10 in situ *hybridisation was performed to assess ectopic *sox10 *expression. A, A' embryos injected with Hs:notch^ΔEmv^. B, B' embryos injected with Hs:Δβ catenin. C. Embryos of the transgenic line *Tg(-4725sox10:GFP)*^*ba*3 ^were injected with equivalent constructs lacking GFP-coding regions. C, C' embryos injected with hs:zsox9b. A-C no heatshock, A'-C' with heatshock. Ectopic *sox10 *or *gfp *expression is indicated by the coloured reaction product (arrows). Note that the low level expression in B reflects the known leakiness of this heatshock promoter [71].

The critical regions of the *sox10 *promoter highlighted in this study contained numerous binding sites for transcription factors previously implicated in both neural crest and *sox10 *regulation, including Notch, β-catenin/Tcf, FoxD3 and SoxE proteins. Before focusing our attention on the highlighted regions we wished to confirm whether these proteins were indeed individually competent to induce *sox10 *expression in zebrafish. Embryos at the single cell stage were injected with constructs containing *notch*^Δ*Emv*^, a constitutively active Notch, Δ*β-catenin *(an activated *βcatenin*) or *sox9b *under the control of a heatshock promoter. The embryos were incubated at 28.5°C for 6 hours before being subjected to a 60 min, 37°C heatshock, then left for 1 hour before fixing and processing to detect *sox10 *transcripts by in situ hybridization. These embryos, equivalent to approximately 8-9 hpf embryos, would be expected to be too young to show endogenous *sox10 *expression at this stage. Consistent with this, uninjected embryos subjected to heatshock did not express detectable levels of *sox10*. In contrast, embryos expressing Sox9b, Notch or β-catenin showed induced *sox10 *transcripts in the expected mosaic pattern (Fig. 6A,B; data not shown). Quantitation indicated that all three transcription factors were able to induce *sox10 *expression in these early embryo tests (Table 3). In contrast, heatshock promoter driven FoxD3 was unable to induce ectopic *sox10 *expression in this experiment (data not shown).

**Table 3:** Notch, βcatenin and SoxE proteins induce *sox10 *transcript expression

	% positive ectopic *sox10 *expression	
	without heatshock	with heatshock

Uninjected	ND	1 (n = 91)
Notch^ΔEmv^	27(n = 33)	54 (n = 41)
βcatenin^Δ^	27 (n = 86)	54 (n = 95)
Sox9b	5 (n = 41)	44 (n = 34)

Zebrafish embryos injected with heatshock plasmid constructs at the 1-cell stage were incubated for 6 hours before a 60 min 37°C heatshock. After a further 1 hour incubation at 28.5°C embryos were fixed and processed for ectopic *sox10 *expression by *in situ *hybridisation.

To support further the idea that regulatory regions within the promoter region analysed here might be responsive to these transcription factors, we repeated the heatshock experiments in the *Tg(-4725sox10:GFP)*^*ba*3 ^reporter line. Embryos were injected with 400 pg of either hs:notch^ΔEmvΔGFP ^(as hs:notch^ΔEmv ^but with GFP coding region deleted) or hs:zsox9b, heat-shocked for 1 hour at 6 hpf, then left for one hour, before being fixed and examined by whole-mount mRNA in situ hybridisation for *gfp *expression. Uninjected embryos showed no *gfp *expression confirming that at the stage examined endogenous *gfp *expression is lacking; in contrast a large proportion of embryos injected with either the *sox9b *or *notch *constructs showed *gfp *expression (Table 4; Figure 6C). Interestingly, the *sox9b *construct showed a significantly increased proportion of embryos with ectopic *gfp*^+ ^in injected embryos after heat shock when compared with non-heat-shocked controls, whereas the *notch *construct did not. Given the known leakiness of this heat shock promoter [71], this result indicates that the transgene is very sensitive to even low levels of Notch activity. Together, these data confirm the responsiveness of the *sox10 *promoter to Sox9b and Notch.

**Table 4:** Notch and SoxE proteins induce *gfp *transcript expression from *sox10:gfp *transgenics

	% positive ectopic *gfp *expression	
	without heatshock	with heatshock

Uninjected	1.2 (n = 78)	0 (n = 112)
Notch^ΔEmvΔGFP^	79 (n = 39)	75 (n = 107)
Sox9b	18.5 (n = 92)	52.5 (n = 154)*

* p < 0.0001 (Chi squared test)

*Tg(-4725sox10:GFP)*^*ba*3 ^embryos injected with heatshock plasmid constructs at the 1-cell stage were incubated for 6 hours before a 60 min 37°C heatshock. After a further 1 hour incubation at 28.5°C embryos were fixed and processed for ectopic *gfp *expression by *in situ *hybridisation.

### Characterisation of regulatory elements in the 3' end of intron 1

**Figure 7:**
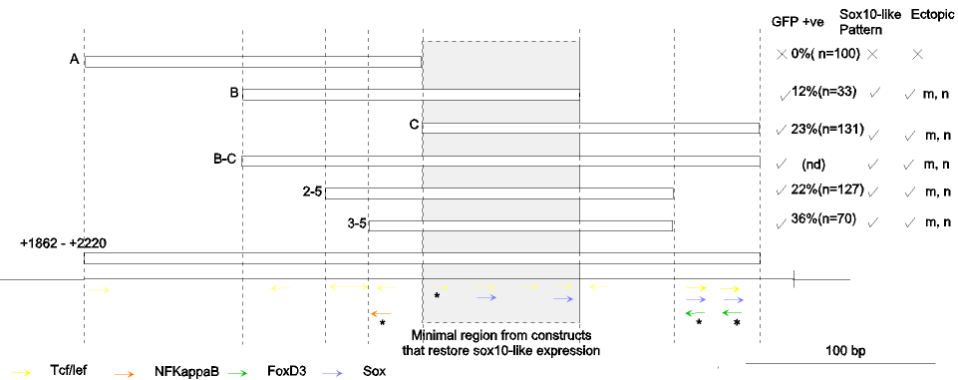
**Sequence at the 3' end of the zebrafish *sox10 *intron 1 is essential for *sox10*:GFP expression.** Cohorts of zebrafish injected with constructs containing *sox10 *regions at the 3' end of intron 1 as shown were used to identify a minimal sequence essential for *sox10*:GFP expression. Intron 1 fragments were replaced into an inactive *sox10*:GFP construct and injected embryos examined for GFP expression as previously described in Fig. 4. The transcription factor binding sites identified by TRANSFAC analysis within this region are illustrated by coloured arrows: red (NFKappaB), yellow (Tcf/Lef), green (FoxD3) and blue (Sox family). The transcription factor binding sites within the sequence that are highly conserved in vertebrate *sox10 *promoter regions are marked (*). m, muscle n, notochord.

Our combined bioinformatics and physical promoter deletion studies had independently converged to indicate that the 3' end of zebrafish *sox10 *intron 1 might contain regulatory sequences critical for *sox10 *expression. To characterise functionally the 376 bp region at the 3'end of intron 1 it was initially divided into 3 overlapping regions (Fig. 7). DNA pieces corresponding to these were generated by PCR and ligated into the p*sox10*^-1252Δ(+1862–+2220)^:GFP vector. This vector lacks the entire 376 bp piece and both transient and germline transgenic fish made with this vector do not express GFP in a *sox10*-like pattern. Re-introduction of fragment A into this vector did not restore any GFP expression. Insertion of fragment B restored the GFP expression, albeit only weakly (in 12% of scored fish; n = 33). Notably, the distribution of these GFP positive cells was consistent with the *sox10*-like expression pattern (see +B in Table 2). Re-introduction of fragment C into the inactive vector also restored GFP to 23% of the fish scored and again the distribution of GFP positive cells largely corresponded to that expected for *sox10 *expression (see +C, Table 2). Significantly, a construct (p*sox10*^-1252Δ(+1862–+2220)+BC^) containing a fragment (BC) spanning both the B and C regions was able to restore GFP expression in appropriate cell types and with a distribution not significantly different (p > 0.05) to the parent p*sox10*^-1252^:GFP construct (see +BC, Table 2). Thus, adding the BC fragment, which spans all six conserved, biologically relevant sequence predictions identified bioinformatically, to the p*sox10*^-1252Δ(+1862–+2220) ^vector is sufficient to restore most aspects of the *sox10*-like pattern. The positions of these putative transcription factor binding sites in the B and C regions are shown in Fig. 7.

To assay the functional relevance of these sites in *sox10*:GFP expression another intronic deletion sequence containing these sites and including the minimal required region was generated. The 2–5 fragment contained multiple sites with invariant CAAA cores of consensus Lef/Tcf/Sox sites(A/T A/T CAA A/T G/T) and includes the evolutionarily conserved Tcf and NFKappaB sites but not the FoxD3/Sox sites identified in the TRANSFAC analysis of *sox10 *promoters. Reintroduction of this piece into an inactive vector (p*sox10*-^1252Δ(+1862–+2220)^) could restore GFP expression in 22% of fish counted and gave a *sox10*-like pattern (see +2–5, Table 2), although the robustness of the *sox10*-like profile was reduced. We concluded that this region with multiple transcription binding sites was sufficient to restore the correct *sox10*-like expression of the heterologous reporter.

### Functional testing of the critical sox10 regulatory region

**Figure 8:**
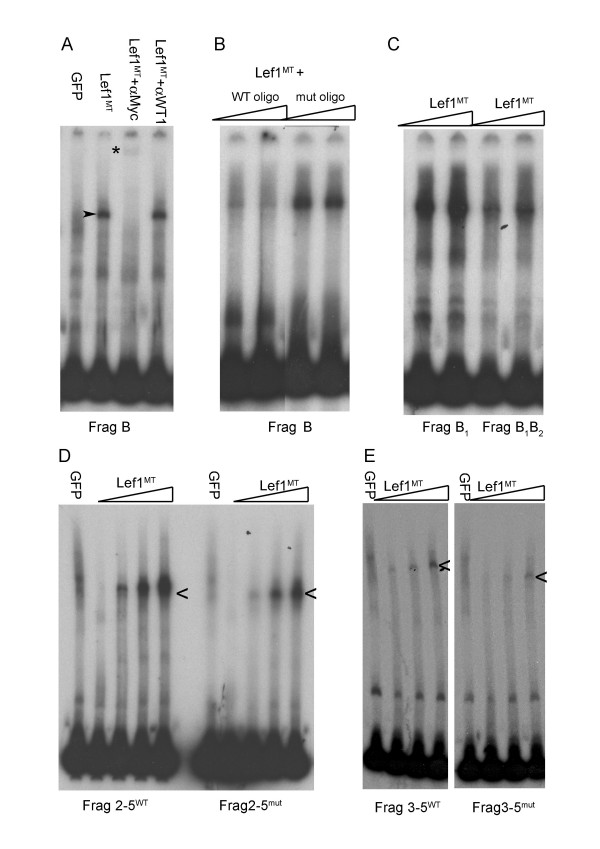
**(A,) Electrophoretic mobility shift assays show specific Lef1 binding to 3' *sox10 *intron 1 DNA sequences contained in Fragment (Frag) B.** A specific band of reduced electrophoretic mobility (arrowhead) is observed when the 3' intron 1 fragment B is incubated with *in vitro *made myc-tagged Lef1 (Lef1^MT^) protein but not when incubated with *in vitro *made GFP protein. The specific Lef1^MT^/DNA complex is lost in the presence of anti-myc antibody and replaced by a supershifted band (*); this band is specific to Lef1^MT ^since this supershift is not seen with an unrelated (anti-WT1) antibody. (B) Competition with excess unlabeled oligonucleotide containing a wild type Lef1 binding site (WT oligo) abolishes the specific binding of Lef1 with Fragment B but oligonucleotide in which the Lef1-binding motif is mutated (mut oligo) is unable to compete for specific binding. (C) Site directed mutagenesis of the conserved Lef1 binding site B1 has little effect on Lef1 binding to Fragment B, additional mutation of site B2 (to give Frag B1,B2) reduces binding of Lef1. (D) Lef1 binding to fragment 2–5 (2–5^WT^) is reduced by mutagenesis of sites B1, B2 and site 5 in 2–5^mut^. (E). Reduced Lef1 binding to fragment 3–5 containing only two conserved Lef1 binding sequences.

Given the robust induction of *sox10 *transcription by β-catenin, and given the prominent role of Wnt signalling in neural crest induction [39,54,72], we wanted to test if the Tcf/Lef binding sites identified in the B and 2–5 fragments could act as functional Tcf/Lef binding sites. Electrophoretic mobility shift assays (EMSA) were performed using radioactively labelled fragments and myc-tagged Lef1^MT ^protein (Fig. 8A). A specific DNA band with reduced mobility was present in reactions containing Fragment B and Lef1^MT ^protein but not in those with GFP protein made *in vitro*. To ensure that the band was not due to the presence of rabbit reticulocyte extract a supershift assay was performed with anti-Myc antibody. In the presence of the anti-Myc tag antibody the specific retarded DNA complex was abolished and replaced by a supershifted band (*, Fig. 8A). The specific complex was however unaffected by incubation with an unrelated anti-WT1 antibody. Competition assays with unlabelled double stranded oligonucleotides showed that an oligonucleotide containing a wild-type Lef1 binding site (GTCAAAG) could abolish the specific binding of Lef1^MT ^to fragment B but oligonucleotides containing only a mutated site (AGCTGAG) were unable to compete for specific binding (Fig. 8B).

To directly address whether the Lef1 binding sites might be crucial for *sox10 *regulation, we next used site directed mutagenesis (Table 5) to disrupt the CAA core of the conserved Lef1 binding sites B1, B2 and site 5 (see Figure 7). Mutation of site B1 alone had little effect on Lef1 binding *in vitro *(Frag B1, Fig 8C) but additional mutation of site B2 reduced Lef1 binding to a fragment carrying both mutations (Fragment B1B2, Fig 8C). Fragment 2–5, previously shown to restore sox10:GFP spatial expression in transient transgenics, also efficiently bound Lef1 in mobility shift assays (Fig 8D) and mutagenesis of the sites B1, B2 and site 5 in this fragment also reduced but did not eliminate Lef1 binding (Frag 2–5^mut^, Fig 8D). To eliminate residual Lef1 binding to the B1 site we made fragment 3–5. This fragment contains only two of the identified evolutionarily conserved Lef1 binding sites. Fragment 3–5 bound *in vitro *made Lef1 less efficiently than fragment 2–5 (Fig 8E) but insertion of this fragment into the inactive vector was still able to restore sox10:GFP expression (18/70 embryos screened at 48 hpf). While this result further delineates the regulatory region required for *sox10*-like reporter expression to that surrounding the evolutionarily conserved transcription factor binding sites identified in our sequence comparison, mutagenesis of the conserved Lef1 target sites in the 3–5 sequence could not eliminate Lef1 binding (Fig. 8E) or sox10:GFP reporter expression (not shown), indicating that additional target sequences in this region contribute to embryonic *sox10 *expression; these additional sequences may include non-canonical Lef-binding sites, as well as sites binding other transcription factors. Taken together, our data provides evidence that regulation of early *sox10 *expression is complex, and is likely to depend on a combination of both Lef1 and other transcription factors binding to a defined region of intron 1 of *sox10*.

**Table 5:** Mutagenesis of Tcf1/Lef1/SoxE sites in the 2–5, 3–5 fragments consensus motif ^*A*^/_*T*_^*A*^/_*T*_CAA^*A*^/_*T*_^*G*^/_*C*_

**Site B1**	***G*T CAA AG**	**mutated to**	***G*T AGA CA**
B2	A*G *CAA AG	mutated to	A*G *C**TG **AG
5	T*G *CAA AG	mutated to	*T*G CA- --

## Discussion

In this study we have combined comparative sequence analysis and promoter deletion approaches to identify evolutionarily constrained regulatory motifs likely to control expression of *sox10 *in embryos. We determined that transient transgenic zebrafish containing isolated *sox10 *regulatory regions including less than 3.5 kb of genomic DNA sequences 5' of the first zebrafish *sox10 *codon were sufficient to drive GFP expression, both in transient transgenics and in germ-line transgenic animals, in patterns consistent with that previously established for early embryonic *sox10 *expression [8,19]. GFP expression was initiated at the appropriate time in premigratory crest and otic epithelium and reporter expression was maintained in the correct cell types, including neural crest derivatives, the developing ear and later in differentiating oligodendrocytes. This established that in these assays sequences from the Sox10 protein coding region and 3' genomic sequences were not required for initial spatial and temporal *sox10 *expression in zebrafish. Recent promoter analyses in mouse have indicated that the presence of long range enhancers, tens of kilobases distal to the coding and proximal promoter regions, are additionally necessary for correct temporal and spatial gene regulation of both *sox10 *and *sox9 *genes in mice [27,34,60,61]. These mouse studies suggest that additional elements regulating the level of *sox10 *expression are likely to reside outside the 7 Kb of 5' genomic *sox10 *sequence in our zebrafish constructs. Indeed, germline transgenic zebrafish made with the 7 Kb zebrafish *sox10 *promoter element driving a *sox10 *cDNA were only partially able to restore the pigment pattern of *sox10 *mutant fish (JRD, Ben Steventon and RNK, data not shown).

Nevertheless, we show here that the proximal promoter fragments tested in this study do regulate reporter gene expression in a manner recapitulating the early expression pattern of endogenous zebrafish *sox10*. Thus, signals controlling *sox10 *expression in the zebrafish neural crest, developing ear and later in the oligodendrocyte lineage are likely to act via regulatory elements contained in the discrete promoter fragments tested in this study. As deletion of 5' sequence to within 100 bases 5' of the transcript start maintained a *sox10*-like reporter expression pattern in transient transgenic embryos, we hypothesised that this regulation was mediated through elements residing in the 5' untranslated region encoded by exons 1 and 2, or the intervening intron. Further dissection of this region indicated that the key elements for the spatial regulation are contained within the 1.5 kb intron 1.

*Sox10 *sequences are available from diverse vertebrate species and an intron in this position is conserved in all cases. Deletion of the entire intron 1 sequence from the zebrafish *sox10*:GFP promoter constructs almost completely removed transgene expression and resulted in the loss of any discrete *sox10*-like pattern of GFP expression. As shown here, the promoter prediction program FirstEF predicted a core promoter overlapping the first intron of *sox10 *in all species analyzed. These data combined are consistent with intron 1 sequences being critical for the enhancement and/or initiation of *sox10*:GFP expression. Regulatory elements within intronic sequences have been identified in many other genes including *sox9 *[73] with cases where the intronic context is also essential [74-76]. The importance of evolutionarily conserved regulatory elements within vertebrate introns is clearly recognised [77]. It is less clear how to interpret the highly-conserved promoter prediction in intron 1, and the lack of promoter prediction associated with sequences directly upstream of the *sox10 *transcription start site. One possibility is that intron 1 of *sox10 *contains an enhancer that is necessary to obtain visible levels of GFP expression in our assays. Another possibility is that *sox10 *has a second transcription start site generating a class of *sox10 *transcripts associated with a downstream, or intronic, promoter and certain mRNA and EST data in human and mouse support this notion (AA, data not shown). However, we have no empirical evidence that this promoter is utilized in zebrafish. A transcript start has previously been identified by 5' RACE [8](A. Pauliny, PhD Thesis, University of Bath; TJD, PhD Thesis, University of Bath), using nested primers that were located towards the 3' end of exon 2 in the region that encodes the HMG box DNA binding domain. The nested 5' RACE of cDNA derived from pooled zebrafish embryos resulted in a single band on agarose gels of a size consistent with the inclusion of both exon 1 and exon 2 sequences. This was confirmed by cloning and sequencing the amplified products and it was noted that the sequences terminated at a guanine residue not found in the genomic *sox10 *sequence and, therefore, presumed to represent the 5' cap. We cannot exclude the possibility that the predicted intronic promoter is utilized at low levels or at developmental stages not represented in our 5' RACE experiments. The positioning of the 5' RACE primers would allow for the amplification of transcripts encoding the highly conserved HMG domain, including any that might originate from a promoter within intron 1. However, the only transcripts identified were those that start in a position that is consistent with the known intron-exon structure of *sox10 *and that is conserved in several vertebrate species. Together with our deletion analysis of sequences 5' of exon 1, this suggests that the regulatory elements identified within the 3' region of intron 1 most likely represent enhancer, rather than promoter, sequences, at least in zebrafish.

Following deletion of a 309 bp 5' portion of *sox10 *intron 1, GFP expression in oligodendrocytes was significantly reduced but expression in pigment cells, ear epithelium, nasal cells or facial and pectoral fin cartilage was unaffected. Sequence analysis showed the presence of a high-affinity CSL binding site TGTGGGAA (consensus YGTGDGAA) within the deleted sequence. CSL is the nuclear effector component mediating Notch signalling. Both Notch signalling and expression of *sox10 *have been shown to be necessary for the differentiation of oligodendrocyes in the embryonic neural tube [12,59]. A single low affinity CSL binding site resides just 5' of the deletion and further experiments will be required to determine if the combined deletion of both CSL sites might prevent Notch-dependent regulation of *sox10 *expression in the oligodendrocyte lineage.

Significantly, however, it is the 3' part of the *sox10 *intron 1 that our bioinformatics and functional analysis have independently identified as critical for *sox10*:GFP expression. Although initial analysis did not identify significant non-coding sequence conservation between zebrafish and mammalian *sox10 *genes [61]; this study), the focused approach used here, centred on the experimentally validated regulatory region, identified short sequences highly conserved in *sox10 *intron 1 of mammals, chicken and zebrafish. Strikingly, these included putative regulatory sequences known to bind transcription factors, such as Tcf/Lef, Sox and FoxD3, previously suggested as candidates controlling the expression of various neural crest genes including *sox10 *in premigratory neural crest [38,39,45-47,50-53]. We show that the arrangement of these motifs is conserved in chicken and zebrafish *sox10 *intron 1, highlighting the possible evolutionary conservation of critical regulatory elements for this gene. Strikingly, sequence analysis of these binding sites in the critical region of the zebrafish *sox10 *promoter shows a great number of clustered, closely related Tcf/Lef binding sites some of which overlap with the Sox and FoxD3 motifs. This is interesting because the context and exact sequence of transcription factor target motifs has been shown to regulate the binding affinity of the target Tcf factors [78] and competition for binding to overlapping sequences may also contribute to controlling the exact expression profile of *sox10 *in the developing zebrafish embryo. We begin to dissect the roles for these transcription factors in regulating *sox10 *transcription and show that mutagenesis of several Tcf/Lef binding sites in a minimal region is insufficient to abrogate transcriptional activity of this region, suggesting that regulation is complex. While our in vivo data is consistent with direct regulation of *sox10 *by Lef factors, a view supported at least in part by our initial mutagenesis analysis of the minimal regulatory region we identified, our in vivo data also indicates regulation by Sox9b and Notch; further studies will be required to prove whether regulation of *sox10 *by each of the three factors identified here is direct or indirect.

## Conclusion

The mutational analysis initiated in this study indicates that *sox10 *regulation by this intron 1 region is complex, and depends only in part upon Lef binding sites. It will be interesting to use chromatin precipitation profiling to identify the protein complexes residing on each of these regulatory motifs at different stages of embryonic development as *sox10 *expression changes. Further mutational analysis might reveal the relative importance of the identified sites, but our data indicates that regulation is likely to depend on multiple sites within the critical region identified in intron 1, with these acting at least partially redundantly to drive *sox10 *expression during embryonic zebrafish development. A recent analysis of mouse *Sox10 *regulation identified multiple partially redundant conserved regulatory regions distal to the *Sox10 *promoter and concluded that proximal regions were less important for regulation in mouse [61]. Our data confirm and extend the evidence that proximal regions, particularly those in intron 1, are important for correct *sox10 *regulation in zebrafish [13,19]. Interestingly these studies in both zebrafish and mouse implicate the same key regulatory factors in controlling *sox10 *expression. Further work will be needed to identify whether distal and proximal regulatory elements function redundantly in both species or are divergent evolutionary solutions to the problem of conservation of *sox10 *regulation.

## Methods

### Fish Husbandry

Embryos were obtained through natural mating and maintained at 28.5°C. Embryonic stages are as described by Kimmel et al. [79]. Embryo ages are described in hours post-fertilization (hpf).

### Construction of zebrafish sox10 promoter constructs

A PAC clone (BUSMP706I16137Q2) with an 84 Kb insert encompassing the zebrafish Sox10 coding region was partially sequenced 5' and 3' of the previously identified *sox10 *coding region (GenBank RefSeq ID NM_131875; TJC, PhD Thesis University of Bath) (Fig. 1A and 1B). This sequence enabled both sub-cloning of potential zebrafish *sox10 *promoter sequences and a bioinformatics comparison of the zebrafish *sox10 *5' UTR with potential *sox10 *promoter sequences from other vertebrates. Plasmid p*sox10*^-4725^:GFP (Figs 1 and 4) was constructed as follows. A 7 kb *Eco*R1-*Spe*I fragment terminating at the 3' end just prior to the ATG of the zebrafish *sox10 *gene was isolated from BUSMP706I16137Q2 (TJC, PhD. Thesis University of Bath) and cloned into *Eco*RI-*Spe*I digested pBluescript KS+ (Stratagene). The fragment was subsequently released as a *Sal*I-*Xba*I fragment and cloned into *Sal*I-*Xba*I digested pCS2XLTGFP to form p*sox10*^-4725^:GFP. Note that in this manuscript we number all constructs with respect to the transcription start site (+1); the p*sox10*^-4725 ^promoter fragment corresponds to the promoter fragment used in previous publications [13,67]. Plasmids p*sox10*^-1252^:GFP and p*sox10*^-521^:GFP were created by double-digesting p*sox10*^-4725^:GFP with *Eco*RI and *Mlu*I, and with *Eco*RI and *Nde*I, respectively. Sticky ends were rendered blunt with T4 DNA polymerase and plasmids recircularised using T4 DNA ligase. Plasmid p+20:GFP was described by TJC (PhD. Thesis, University of Bath). Constructs p*sox10*^-1000^cDNA and p*sox10*^-1252Δ(+133–2238) ^were constructed by PCR using annealed overlapping fragments generated from a *sox10 *5' RACE clone [8] and p*sox10*^-4725^:GFP. Site directed mutagenesis (Quik Change XL, Stratagene) was used to insert restriction sites for further deletions: *Nhe*I at position -100, *Eco*RV at +159, *Sma*I at +2220. Deletions between engineered and naturally occurring sites were used for further deletion constructs: p*sox10*^-100^:GFP was formed by re-ligation between *Nhe*I at -100 and *Eco*RI at -4725. Other deletions were: Δ+159 – +1862 re-ligated between *Eco*RV and *Sma*I, Δ+1862 – +2220 re-ligated between *Sma*I sites, Δ+159 – +468 re-ligated beween *Eco*RV and *Bmg*B1 and Δ+468 – +1862 re-ligated between *Bmg*BI and *Sma*I. Restriction analysis and sequencing were used to confirm vector construction. Prior to embryo injection all plasmids were isolated using a High Purity Maxi-prep kit (Marlingen), DNA concentration was determined by spectrophotometry and confirmed by agarose gel electrophoresis.

### Expression Constructs

The hs:notch^ΔEmv^-hs:GFP plasmid encodes mNotchΔE, a Notch mutant which undergoes ligand-independent proteolytic cleavage, under the control of the heatshock promoter. The plasmid also encodes GFP under the control of a second HS promoter and was a gift from Dr Caroline Beck. To test this construct in the *Tg(-4725sox10:GFP)*^*ba*3 ^transgenic line, we deleted the GFP coding region by double digestion with NdeI/SpeI followed by religation, to make plasmid hs:notch^ΔEmvΔGFP^. pCH85 encoding hs:Δβ-catenin was provided by Arne Lekven. Hs:Fkd6 plasmid was constructed by cloning the zebrafish Fkd6 coding region as a BamHI-XhoI fragment into pCsHsp. The zebrafish Sox9b coding region was cloned as an XbaI fragment into pCsHsp from a plasmid supplied by John Postlethwait (University of Oregon, USA) to generate plasmid hs:zsox9b. Myc-tagged Lef1 was made from pCs2Lef1^Myc^[37], kindly provided by Richard Dorsky (University of Utah, USA).

### Generation and scoring of transient transgenic embryos

10 nl purified, undigested plasmid DNA at a concentration of 40 ng/μl in water containing 0.1% phenol red was injected into the yolk of 1 cell stage fertilized, wild-type zebrafish embryos. Morphologically abnormal embryos were identified and discarded at 24 hpf. GFP expression was initially observed in live embryos using a MZFL fluorescent dissecting microscope (Leica) before confirmation and documentation using an Eclipse E800 compound microscope (Nikon). GFP expressing cells were identified by their location and morphology and cohorts of at least 35 GFP-positive embryos were examined in detail to compile an overall expression profile for each construct, paying special attention to known *sox10 *expression domains. Photographic documentation utilised a C4880 CCD camera (Hamamatsu) and compiled using Adobe Photoshop.

### Generation and scoring of GFP germline transgenic lines

*sox10 *promoter:GFP germline transgenic zebrafish lines were generated by co-injection of plasmid constructs containing *I-Sce*I restriction sites and *I-Sce*I restriction enzyme (Roche). Oligonucleotides containing *I-Sce*I restriction sites (TAGGGATAACAGGGTAAT) and appropriate flanking sequences were inserted into *sox10 *promoter constructs at the *HindIII *and *Asp718 *sites flanking the transgene. 10 nl purified, undigested plasmid DNA at a concentration of 10 ng/μl was injected into the cell cytoplasm of 1-cell stage fertilized embryos in injection mix containing 0.5× Mg free *I-Sce*I buffer and 0.2 units/μl *I-Sce*I (Roche, aliquoted and stored at -80°C). Injected embryos with mosaic GFP expression were identified after 24 hpf and the individuals with the highest contribution of GFP-positive cells (approximately 30% of GFP positive embryos) were raised to adulthood. Subsequent crossing with wild-type fish enabled identification of founder germline transgenic fish for individual lines following observation of non-mosaic GFP expression in F1 embryos. Temporal and spatial expression of GFP was monitored and documented as for the transient transgenic embryos. Additional images were taken using a LSM 510 (Zeiss) confocal microscope.

### Promoter and transcription factor binding site predictions

Genomic sequences were obtained by sequencing bacterial artificial chromosomes harboring the *sox10 *locus or from the University of California at Santa Cruz (UCSC) Genome Browser  as previously described (Antonellis et al. 2006): human (chr22:36606845–36725000, July 2003 UCSC assembly), cat (GenBank AC137542), dog (GenBank AC137537), cow (GenBank AC137534), pig (GenBank AC137657), rat (GenBank AC137528), mouse (chr15:79203055–79300747, March 2005 UCSC assembly), chicken (GenBank AC147863) and zebrafish (see above). Genomic sequences corresponding to the 5' region of *sox10 *from each species were submitted to the FirstEF web-based software [70] to predict promoters and adjacent first exons. Default FirstEF parameters  were employed. Sequences from intron 1 that were identical in 7 mammalian species [27] were submitted to TRANSFAC [80] version 8.1 using the Matrix Search for Transcription Factor Binding Sites (MATCH) and Pattern Search for Transcription Factor Binding Sites (PATCH) interfaces. PATCH parameters were set to identify TRANSFAC entries: (1) in vertebrate genes and for vertebrate transcription factors: (2) six basepairs or greater; and (3) with the maximum number of mismatches set at zero. MATCH parameters were set to identify TRANSFAC entries using the "minimize false negatives" setting. The entire zebrafish and chicken intron 1 sequences were analyzed in the exact same manner.

### Ectopic gene expression in zebrafish embryos

Zebrafish embryos were injected with 10 nl of a heatshock plasmid construct at 40 ng/μl concentration at the 1-cell stage before incubation for 6 hours at 28.5°C. The embryos were then transferred to pre-warmed embryo media for 60 min at 37°C. After a further 1 hour incubation at 28.5°C embryos were fixed and processed for ectopic *sox10 *or *egfp *expression by in situ hybridization [8].

### Electrophoretic mobility shift assay

PCR generated target probes were end labeled with γ^32^P ATP using T4 polynucleotide kinase (New England Biolabs) and purified by native PAGE. In vitro generated protein was made from purified plasmid vectors using TNT coupled transcription/translation systems (Promega) according to the manufacturers instructions. Protein production was confirmed by Western blotting. For electrophoretic mobility assays a 20 μl reaction mixture containing *in vitro *made protein, 2000 cpm labeled probe, 1 μg poly(dI-dC)-poly(dI-dC) (Sigma), 50 mM NaCl, 10 mM Hepes pH7.9, 5 mM MgCl_2_, 0.5 mM EDTA, 3% Ficoll, 0.1 mM DTT and 1 mg/ml BSA was incubated on ice for 20 minutes before separation on a 5%^w^/_v _polyacrylamide (37:1), 0.5% TBE gel at 200 V at 4°C. Dried gels were exposed to Biomax MS X-Ray film (Kodak). In supershift experiments 20 μg antibody (anti-WT1 C19 (SantaCruz, USA), anti-Myc (gift of Jonathan Slack, University of Bath, UK) was added to the reaction tube 5 minutes prior to addition of the labelled probe.

### Statistics

Chi squared tests were employed using Yates' correction. Bonferroni correction was used to allow for multiple comparisons.

## Abbreviations

BSA: bovine serum albumin; EDTA: ethylenediaminetetraacetic acid; GFP: green fluorescent protein; PAGE: polyacrylamide gel electrophoresis.

## Authors' contributions

JRD carried out the zebrafish and molecular genetic studies, participated in the in silico studies and drafted the manuscript. AA carried out the in silico studies and drafted the manuscript. TJC participated in the analysis of the transgenic expression patterns and drafted the manuscript. FR carried out some of the zebrafish studies. WJP, AW and RNK conceived of the study, and participated in its design and coordination and helped to draft the manuscript. All authors read and approved the final manuscript.

## Supplementary Material

Additional file 1 **TRANSFAC analysis of mouse, chicken and zebrafish *sox10 *intron 1.** The sequence of intron 1 for each species was submitted to the TRANSFAC transcription factor binding site database. Shown, in each case, is the 3'-most region of intron 1, with the last two nucleotides of the intron noted in bold italics. Sequences that are highly conserved in mammals are underlined in the mouse intron 1 sequence, and the individual transcription factor binding sites are highlighted in colour. Note the conservation of the order of predictions for (5' to 3') NFKappaB, TCF, FoxD3, and Sox binding-site consensus sequences. Furthermore, with one exception (FoxD3 and Sox consensus sequences in chicken), the orientation of each predicted binding site is also conserved among the three species. Click here for file
