# Supplementary figures and images for: An evolutionarily conserved intronic region controls the spatiotemporal expression of the transcription factor Sox10
Source: BMC Dev Biol. 2008 Oct 26;8:105. doi: 10.1186/1471-213X-8-105 (PMC2601039; doi:10.1186/1471-213X-8-105)

A

Mouse Intron 1

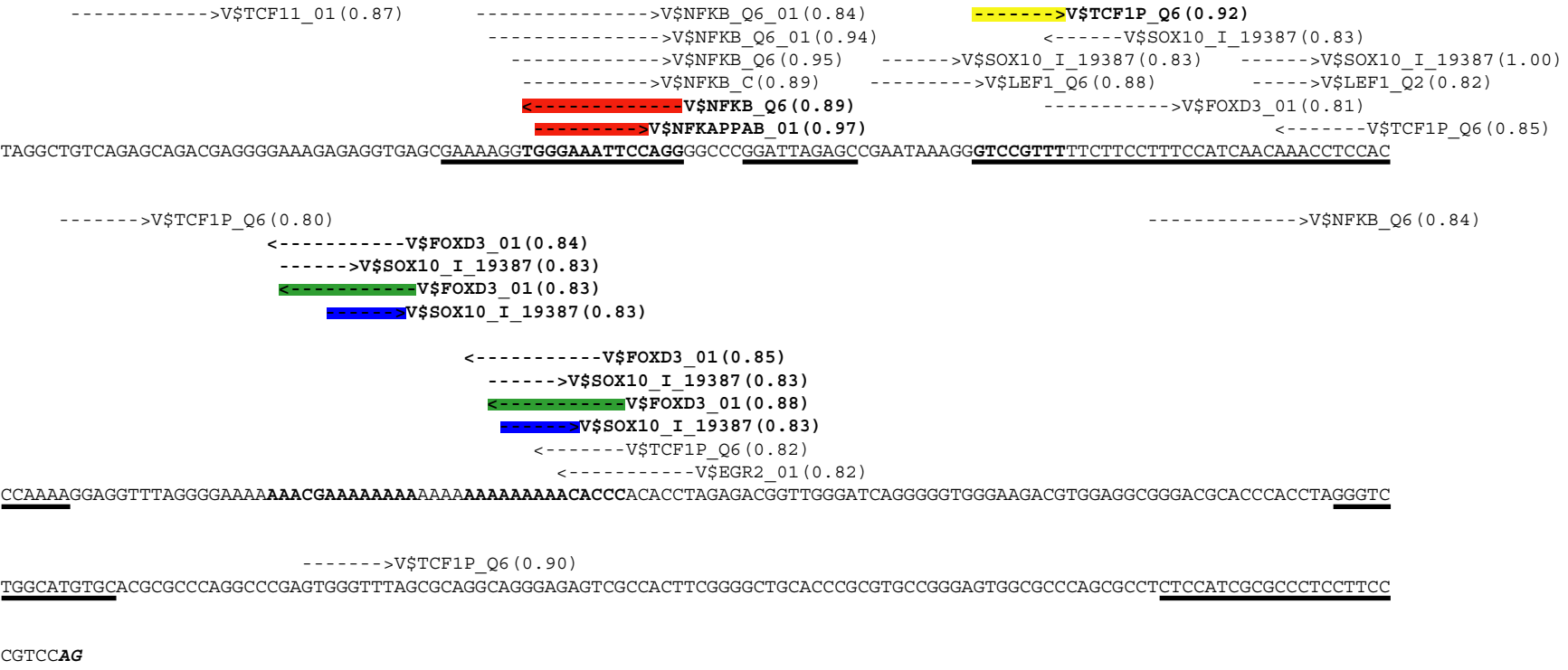

B

Chicken Intron 1 (3' region)

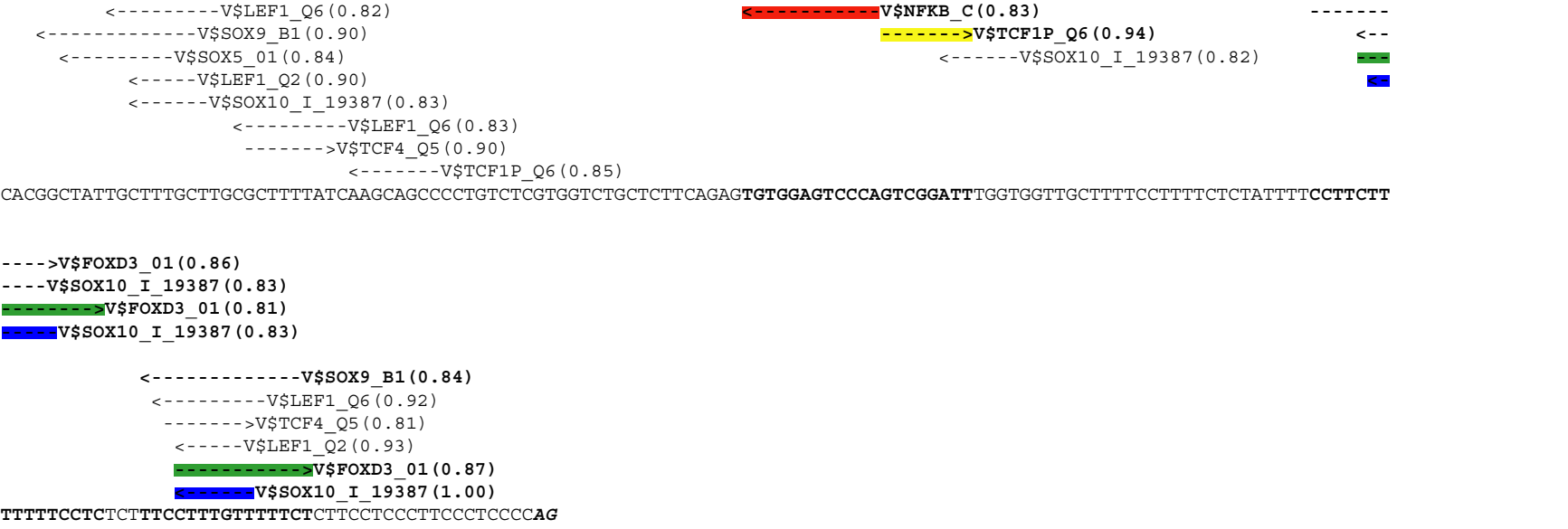

C

Zebrafish Intron 1 (3' region)

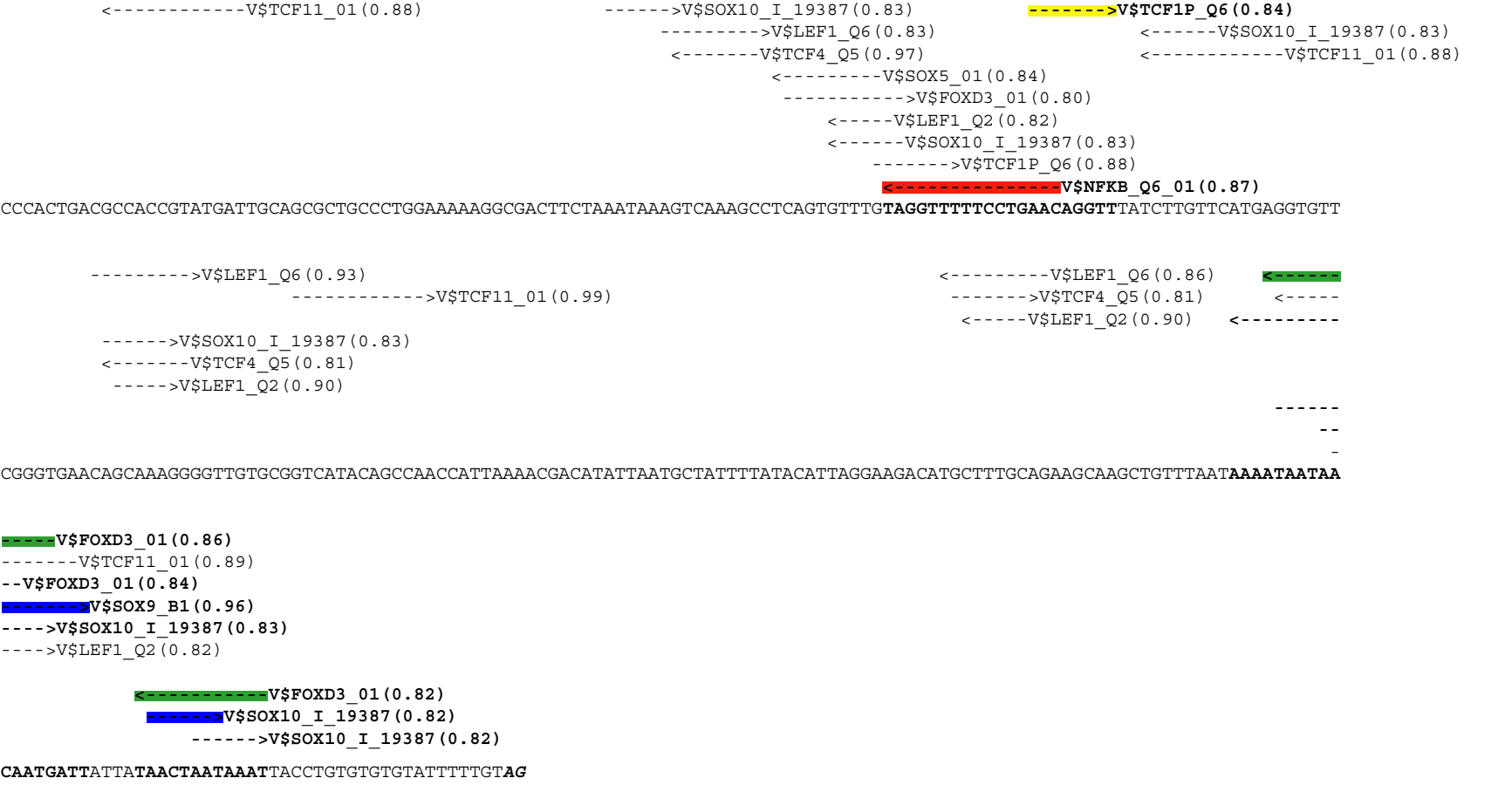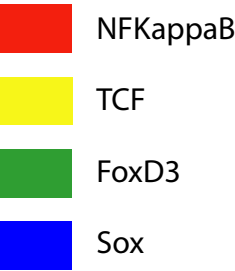

Supplement: Additional file 1 — TRANSFAC analysis of mouse, chicken and zebrafish sox10 intron 1. The sequence of intron 1 for each species was submitted to the TRANSFAC transcription factor binding site database. Shown, in each case, is the 3'-most region of intron 1, with the last two nucleotides of the intron noted in bold italics. Sequences that are highly conserved in mammals are underlined in the mouse intron 1 sequence, and the individual transcription factor binding sites are highlighted in colour. Note the conservation of the order of predictions for (5' to 3') NFKappaB, TCF, FoxD3, and Sox binding-site consensus sequences. Furthermore, with one exception (FoxD3 and Sox consensus sequences in chicken), the orientation of each predicted binding site is also conserved among the three species. [file 1471-213X-8-105-S1.pdf]
